# Knockdown of lncRNA TapSAKI alleviates LPS-induced injury in HK-2 cells through the miR-205/IRF3 pathway

**DOI:** 10.1515/med-2021-0204

**Published:** 2021-04-07

**Authors:** Xiaoning Han, Zhiyong Yuan, Yajun Jing, Weigui Zhou, Yunbo Sun, Jinyan Xing

**Affiliations:** Department of Critical Care Medicine, The Affiliated Hospital of Qingdao University, No. 16, Jiangsu Road, Qingdao 266003, Shandong, China

**Keywords:** sepsis, TapSAKI, miR-205, LPS, cell injury

## Abstract

Sepsis is a common and lethal syndrome. Long non-coding RNA (lncRNA) transcript predicting survival in AKI (TapSAKI) has recently been found to serve as an important regulator in sepsis. However, the underlying mechanism of TapSAKI in sepsis pathogenesis remains largely unknown. Our data demonstrated that lipopolysaccharide (LPS)-induced HK-2 cell injury by weakening cell viability and enhancing cell apoptosis and inflammation. TapSAKI was upregulated and miR-205 was downregulated in LPS-induced HK-2 cells. TapSAKI knockdown or miR-205 overexpression alleviated LPS-induced cytotoxicity in HK-2 cells. TapSAKI sequestered miR-205 via acting as a miR-205 sponge. Moreover, the mitigating effect of TapSAKI silencing on LPS-induced HK-2 cell injury was mediated by miR-205. Additionally, the interferon regulatory factor 3 (IRF3) signaling was involved in the regulation of the TapSAKI/miR-205 axis on LPS-induced HK-2 cell damage. Our current study suggested that TapSAKI silencing relieved LPS-induced injury in HK-2 cells at least in part by sponging miR-205 and regulating the IRF3 signaling pathway, highlighting a novel understanding for sepsis pathogenesis and a promising target for this disease treatment.

## 1 Introduction

Sepsis is a dysregulated systemic inflammatory response to microbial infection that causes organ injury, lean tissue wasting, and death. Although outcomes have improved, the mortality of sepsis is still higher than 30% and even 40–50% when the shock is present [1,2]. Thus, a better understanding of sepsis pathogenesis is meaningful to develop and deploy novel and improved therapeutic agents.

Long non-coding RNAs (lncRNAs) are longer than 200-nucleotide RNA molecules that perform various functions in a wide variety of crucial biological processes [3]. lncRNAs have recently been found to serve as important regulators in human diseases, including sepsis [4]. For instance, Wu et al. reported that Hox transcript antisense intergenic RNA (HOTAIR) was upregulated in lipopolysaccharide (LPS)-induced sepsis mice, and it enhanced inflammation via activating nuclear factor-κB (NF-κB) signaling [5]. Chen and colleagues demonstrated that metastasis-associated lung adenocarcinoma transcript 1 (MALAT1) promoted cardiac inflammation and dysfunction in sepsis through targeting microRNA (miRNA)-125b and regulating the p38 NF-κB pathway [6]. Wang and colleagues reported that lncRNA small nucleolar RNA host gene 16 (SNHG16) regulated LPS-induced inflammatory response by directly interacting with miRNA-15a/16 [7]. As for transcript predicting survival in AKI (TapSAKI), it was highly expressed in the sepsis model and enhanced cell inflammation and apoptosis in HK-2 cells after LPS stimulation [8]. Nevertheless, the mechanism of TapSAKI in sepsis pathogenesis remains largely undiscovered.

Recently, an increasing amount of evidence has suggested that lncRNAs exhibit major biological functions through the network of the competing endogenous RNA by sponging miRNAs in human diseases [9]. miRNAs are 18–23 nucleotides long, single-stranded RNA transcripts that act as essential players in human diseases, such as sepsis [10,11]. A recent document reported that miR-205 was an independent risk factor for sepsis-induced acute kidney injury, and low miR-205 level predicted shorter survival of this patient cohort [12]. Nevertheless, the influence and mechanism of miR-205 in sepsis are still undefined. A putative target sequence between TapSAKI and miR-205 predicted by LncBase Predicted v.2 software prompted us to examine miR-205 as a potential molecular mediator of TapSAKI in sepsis pathogenesis.

In the current work, we first established the sepsis *in vitro* model using LPS. Subsequently, we explored the function impact of TapSAKI on cell viability, apoptosis, and inflammation in LPS-induced HK-2 cells and the underlying mechanism governing it.

## 2 Materials and methods

### 2.1 Cell culture and LPS treatment

Human renal proximal tubular epithelial cell line HK-2 (ATCC^®^CRL-2190; ATCC, Manassas, VA, USA) was grown in Dulbecco’s-modified Eagle’s medium F12 (Invitrogen, Burlington, ON, Canada) plus 10% fetal calf serum (Bovogen, Australia) at 37°C in 95% O_2_/5% CO_2_ atmosphere. To establish the sepsis *in vitro* model, LPS was procured from Sigma-Aldrich (Buchs, Switzerland) and used to treat HK-2 cells at various concentrations (0, 2.5, 5, and 10 µg/mL) or a final 5 µg/mL concentration.

### 2.2 Transfection of oligonucleotides

For TapSAKI silencing, HK-2 cells at 50% confluence were introduced with 30 nM of small interfering RNA (siRNA) against TapSAKI (si-TapSAKI, 5′-AUUUGCAAGGAUUUGACUCUG-3′), and nontarget siRNA (si-NC, 5′-UUCUCCGAACGUGUCACGU-3′) was used as the negative control. For miR-205 overexpression, 30 nM of synthetic miR-205 mimic (5′-GUCUGAGGCCACCUUACUUCCU-3′) or mimic negative control (miR-NC, 5′-UUCUCCGAACGUGUCACGUTT-3′) was transfected into HK-2 cells. miR-205 knockdown was performed using 30 nM of miR-205 inhibitor (anti-miR-205, 5′-AGGAAGUAAGGUGGCCUCAGAC-3′), with nontarget miRNA inhibitor (anti-NC, 5′-UUUGUACUACACAAAAGUACUG-3′) as a negative group. All oligonucleotides were synthesized by Sangon Biotech (Beijing, China) and transfected into cells using the commercially available Lipofectamine 3000 reagent (Invitrogen) following the manufacturer’s recommendation.

### 2.3 Determination of cell viability

A viability test of cells after LPS stimulation and various transfections was performed using a Cell Counting Kit-8 (CCK-8; Beyotime, Shanghai, China) referring to the producer’s suggestion. Briefly, transfected or untransfected HK-2 cells in 96-well plates were exposed to the indicated concentration of LPS for 48 h. Then, CCK-8 reagent (10 µL each well) was used, and absorbance at an optical density of 450 nm was detected after incubation at 37°C for 2 h using an Infinite M200 Pro microplate reader (Tecan, Grödig, Austria). Cell viability was presented as the percentage each concentration accounted for of the negative control.

### 2.4 Measurement of cell apoptosis

HK-2 cells after various treatments were washed five times with ice-cold PBS. The cells (1.0 × 10^5^) were resuspended in 200 µL of binding buffer containing 2 µL of Annexin V-fluorescein isothiocyanate (FITC) and 4 µL of propidium iodide (PI), referring to the suggestion of the Annexin V-FITC/PI Detection Kit (Sigma-Aldrich). The apoptotic cells were analyzed by a flow cytometer (BD Bioscience, Stockholm, Sweden) with a 488 nm laser.

### 2.5 Western blot

Preparation of cell lysates was carried out using the lysis buffer, as described previously [13]. Approximately 50 µg of total protein from each treatment was resolved on an SDS-polyacrylamide gel and transferred onto the nitrocellulose membranes (Bio-Rad, Kidlington, UK) by electro-blotting. The following primary antibodies were used: anti-Ki67 (ab92742; dilution 1:1,000), anti-Bax (ab32503; dilution 1:1,000), anti-interleukin 6 (anti-IL-6, ab6672; dilution 1:500), anti-tumor necrosis factor-α (anti-TNFα, ab6671; dilution 1:1,000), anti-TANK binding kinase-1 (anti-TBK1, ab227182; dilution 1:2,000), anti-interferon regulatory factor 3 (anti-IRF3, ab25950; dilution 1:500), anti-phosphorylated TBK1 (anti-p-TBK1, ab186469; dilution 1:500), anti-p-IRF3 (ab76493; dilution 1:3,000), and anti-β-actin (ab8226, loading control; dilution 1:5,000) (all from Abcam, Cambridge, UK). The immuno-detected proteins were analyzed by chemiluminescence on a ChemiDoc System (Bio-Rad).

### 2.6 Quantitative real-time polymerase chain reaction (qRT-PCR)

In TapSAKI expression assay, total RNA from HK-2 cells after each treatment was extracted using TRIzol reagent (Invitrogen), referring to the suggestion of manufacturers. cDNA was generated from 500 ng of total RNA using the Prime-Script RT reagent kit (TaKaRa, Dalian, China), then subjected to qRT-PCR using Green PCR Mix (TaKaRa). In miR-205 detection assay, total RNA was obtained using mirVana^TM^ miRNA Isolation Kit (Ambion, Invitrogen), reverse-transcribed using the TaqMan RT Kit (Applied Biosystems, Warrington, UK) and subjected to qRT-PCR using TaqMan MicroRNA Assay Kit (Applied Biosystems). qRT-PCR was performed using the following primers: TapSAKI 5′-AAGAAGTCTGCTTCAGGGGC-3′ (Forward-F) and 5′-CACTCCGTGGGAAAGTCCTC-3′ (Reverse-R), GAPDH: 5′-CACCCTGTTGCTGTAGCCATATTC-3′ (F) and 5′-GACATCAAGAAGGTGGTGAAGCAG-3′ (R), miR-205: 5′-CTTGTCCTTCATTCCACCGGA-3′ (F) and 5′-TGCCGCCTGAACTTCACTCC-3′ (R), U6: 5′-CTCGCTTCGGCAGCACA-3′ (F) and 5′-AACGCTTCACGAATTTGCGT-3′ (R). GAPDH or U6 was used as a housekeeping gene for normalization. The relative levels of TapSAKI and miR-205 were calculated by the 2^−ΔΔCt^ method. Additionally, qRT-PCR products were subjected to agarose gel electrophoresis using the E-Gel Power Snap System (Thermo Fisher Scientific, Waltham, MA, USA).

### 2.7 Bioinformatics

Analysis of the potentially interacted miRNAs of TapSAKI was carried out using online database LncBase Predicted v.2 based on complementary base pairing at http://carolina.imis.athena-innovation.gr/diana_tools/web/index.php?r=lncbasev2/index-predicted.

### 2.8 Dual-luciferase reporter assay

The partial sequence of TapSAKI harboring the miR-205-binding site and the mutant sequence in the target site were cloned into the pmirGLO vector (Promega, Nepean, Canada) to construct TapSAKI wild-type luciferase reporter plasmid (TapSAKI-WT) and mutant-type reporter plasmid (TapSAKI-MUT), respectively. HK-2 cells were introduced with 100 ng of TapSAKI-WT or TapSAKI-MUT, together with 30 nM of miR-NC mimic or miR-205 mimic. After 48 h, Firefly and Renilla luciferase activities were synchronously measured using a Promega Reporter Assay System. Relative luciferase activity was normalized against Renilla luciferase activity and expressed as a percentage of the control.

### 2.9 Statistical analysis

Statistical significance of all tests in different groups was analyzed by Student’s *t*-test (two-sided) or one-way analysis of variance with Tukey’s *post hoc* test. Error bars represented standard deviation. Differences were considered significant when *P* < 0.05.

## 3 Results

### 3.1 LPS weakened cell viability and promoted the apoptosis and inflammation in HK-2 cells

**Figure 1:**
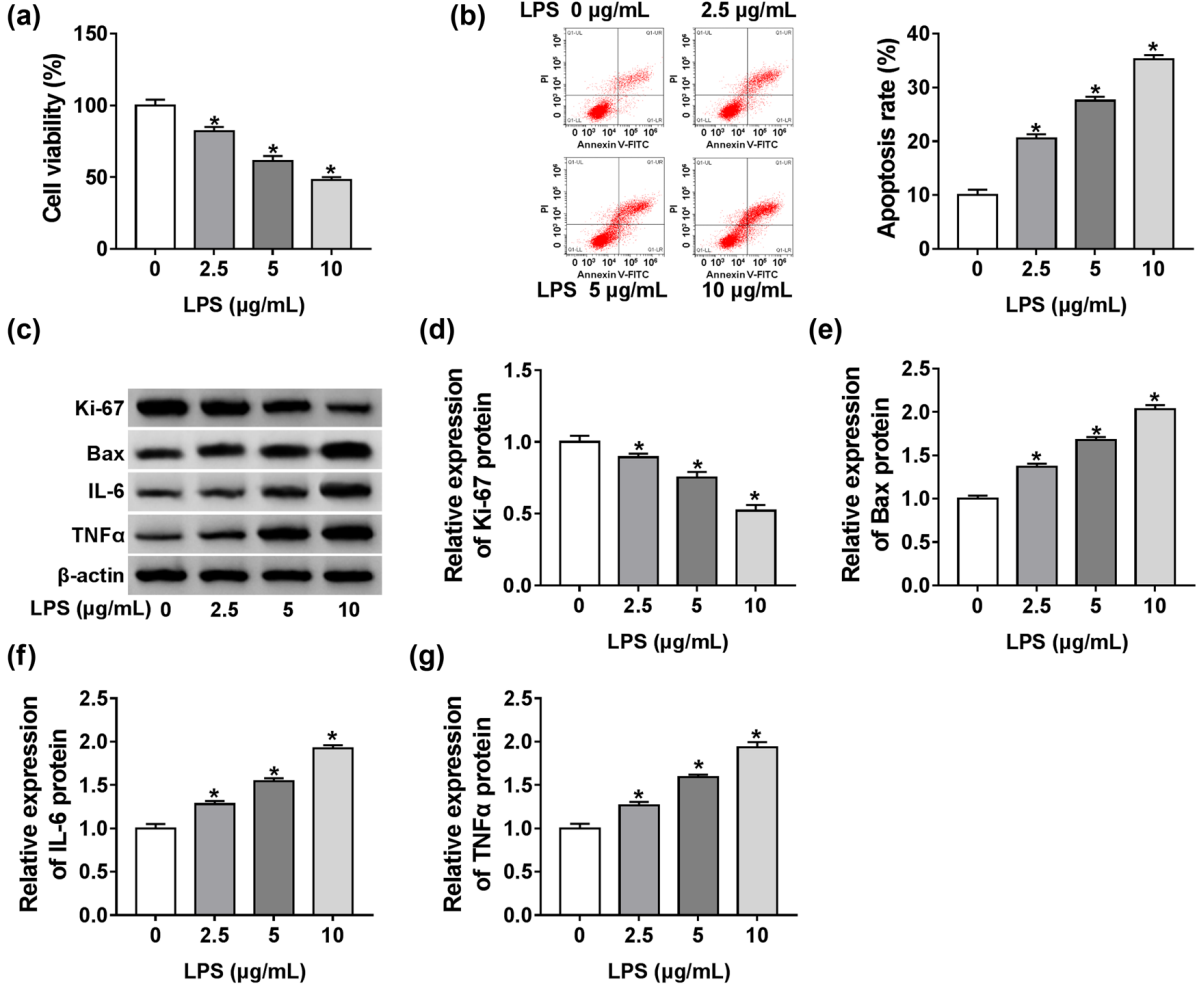
LPS weakened cell viability and promoted apoptosis and inflammation in HK-2 cells. HK-2 cells were exposed to various concentrations (0, 2.5, 5, and 10 µg/mL) of LPS for 48 h. (a) CCK-8 assay for cell viability. (b) Flow cytometry for cell apoptosis. (c–g) Western blot for Ki-67, Bax, IL-6, and TNFα levels. *P < 0.05.

We first evaluated the impact of LPS on HK-2 cell viability, apoptosis, and inflammation. The data of CCK-8 and flow cytometry assays showed that when compared with the negative group, cell viability was significantly hindered (Figure 1a), and cell apoptosis was prominently drove (Figure 1b) by LPS treatment in a dose-dependent manner. Western blot analyses revealed that LPS stimulation resulted in decreased proliferating marker Ki-67 expression and increased pro-apoptosis protein Bax level in HK-2 cells (Figure 1c and e). Moreover, the production of pro-inflammation factors IL-6 and TNFα was dose-dependently elevated by LPS (Figure 1c, f, and g), eliciting the enhancement of LPS on the cellular inflammatory response. All these data suggested that LPS induced injury in HK-2 cells.

### 3.2 TapSAKI was upregulated and miR-205 was downregulated in LPS-treated HK-2 cells

**Figure 2:**
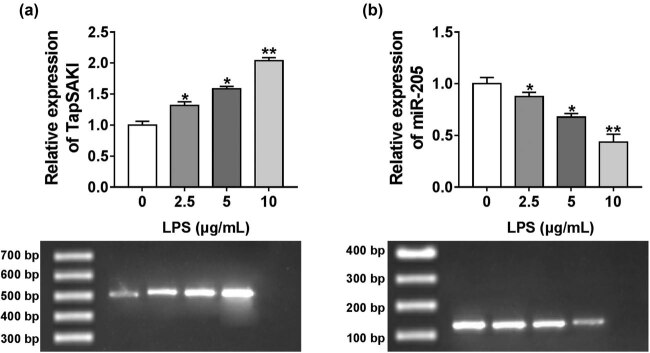
TapSAKI was upregulated and miR-205 was downregulated in HK-2 cells after LPS stimulation. TapSAKI (a) and miR-205 (b) expression levels by qRT-PCR and agarose gel electrophoresis assay in HK-2 cells after stimulation with various concentrations (0, 2.5, 5, and 10 µg/mL) of LPS for 48 h. *P < 0.05, **P < 0.01.

Previous reports found that TapSAKI was closely associated with sepsis-induced kidney injury [8,14]. We then validated the expression of TapSAKI in HK-2 cells after LPS stimulation. As expected, LPS-induced TapSAKI expression in a dose-dependent manner, as compared to the negative control (Figure 2a). More interestingly, the data of qRT-PCR also demonstrated that LPS dose-dependently declined miR-205 expression in HK-2 cells (Figure 2b).

### 3.3 TapSAKI silencing attenuated LPS-induced damage in HK-2 cells

**Figure 3:**
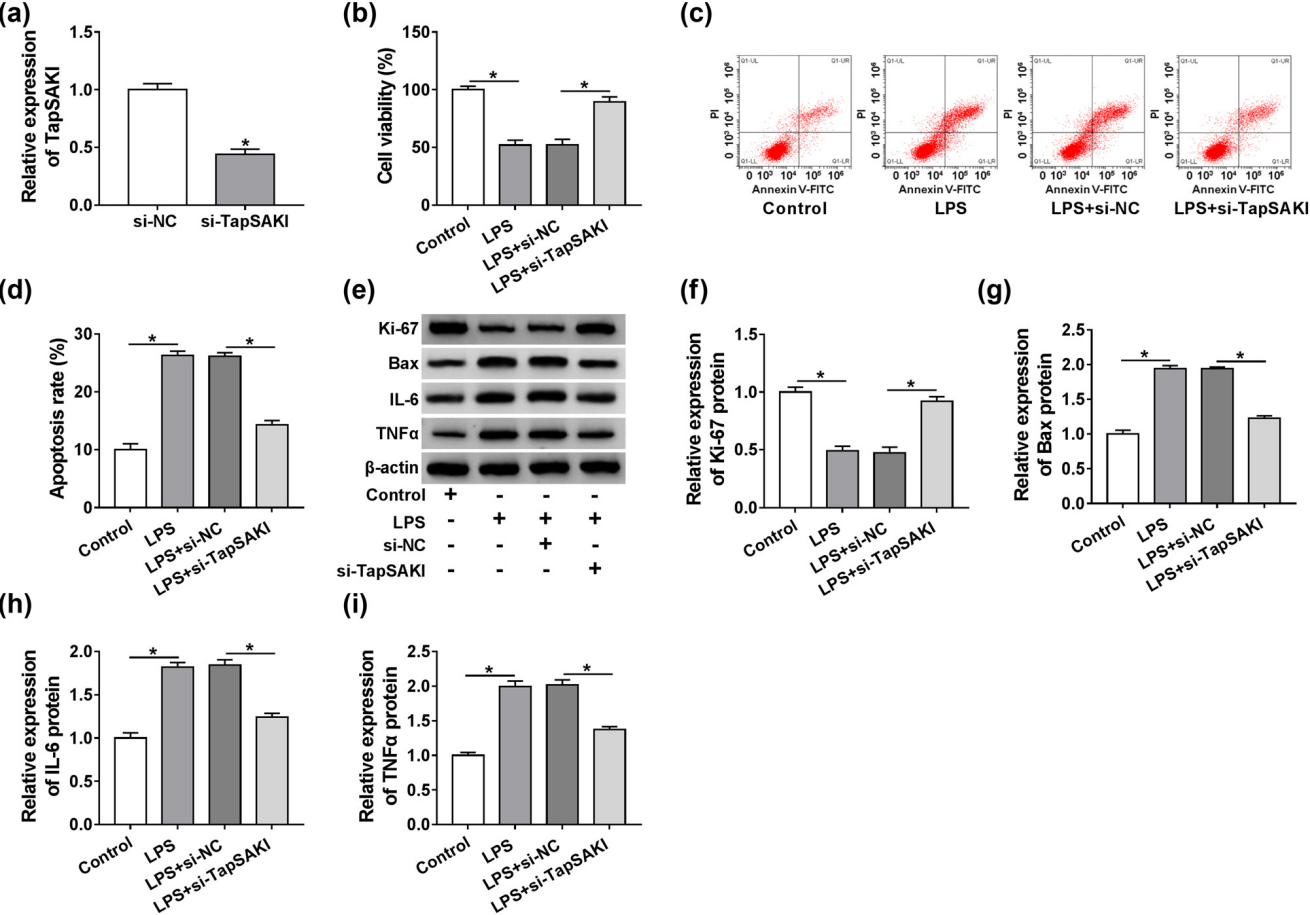
TapSAKI silencing attenuated LPS-induced damage in HK-2 cells. (a) qRT-PCR for TapSAKI expression in HK-2 cells transfected with si-NC or si-TapSAKI. Cell viability by CCK-8 assay (b), cell apoptosis by flow cytometry (c and d), Ki-67, Bax, IL-6, and TNFα levels by western blot (e–i) in HK-2 cells transfected with or without si-NC or si-TapSAKI before LPS exposure. *P < 0.05.

To elucidate the effect of TapSAKI on LPS-treated HK-2 cells, we reduced TapSAKI expression by si-TapSAKI. In contrast to the negative control, the introduction of si-TapSAKI induced about a 59% downregulation of TapSAKI expression in HK-2 cells (Figure 3a), indicating the high transfection efficiency. Subsequent CCK-8 and flow cytometry analyses revealed that LPS-mediated anti-viability (Figure 3b) and pro-apoptosis (Figure 3c) effects were strikingly abolished by TapSAKI knockdown. Moreover, TapSAKI depletion in LPS-treated HK-2 cells led to the restoration of Ki-67, Bax, IL-6, and TNFα levels, which were changed by LPS stimulation (Figure 3d–h). These data together suggested that TapSAKI knockdown protected HK-2 cells from LPS-induced damage.

### 3.4 TapSAKI acted as a sponge of miR-205

**Figure 4:**
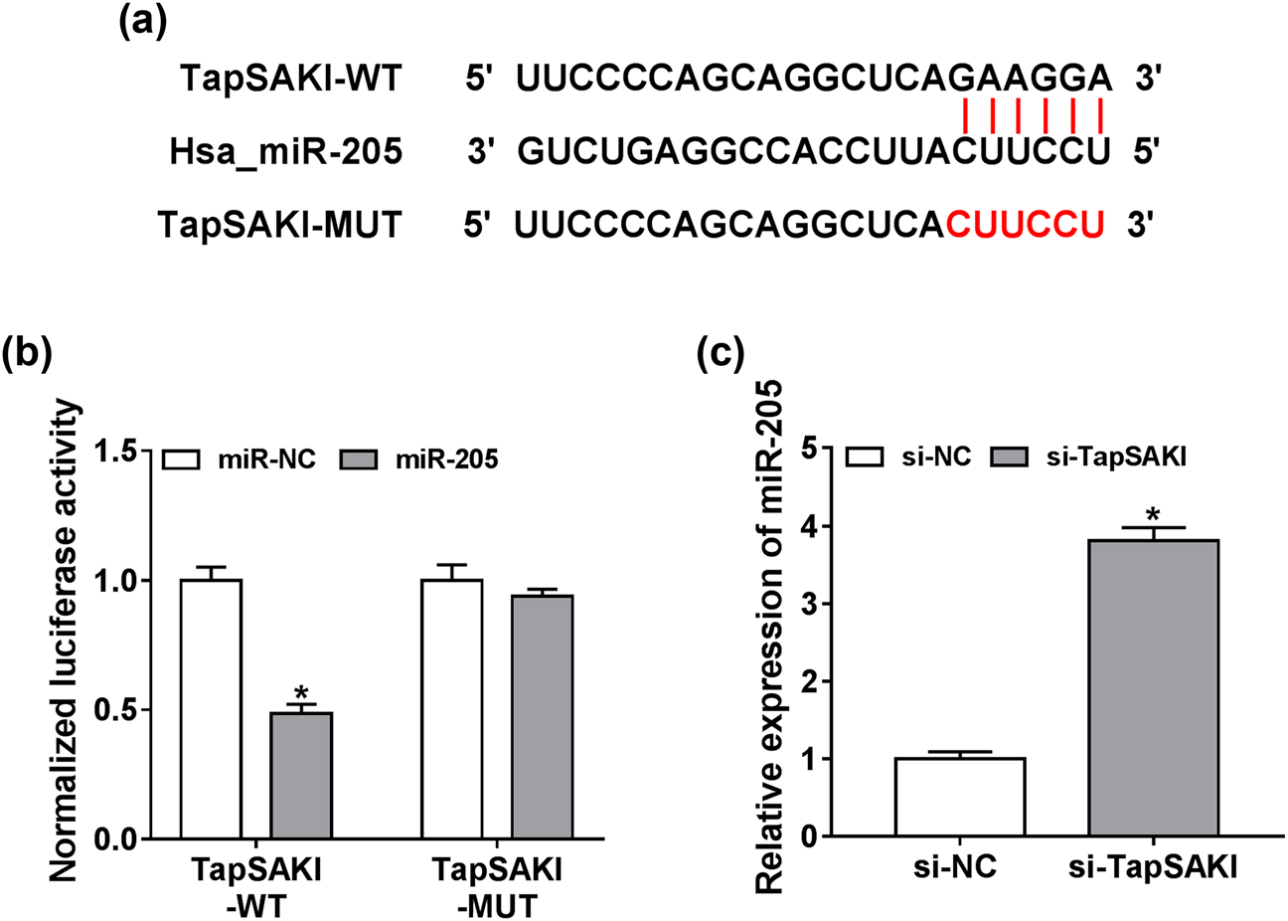
TapSAKI directly interacted with miR-205. (a) Schematic of the complementary sequence for miR-205 in TapSAKI identified by LncBase Predicted v.2 software and the mutant in the target sequence. (b) Dual-luciferase reporter assay in HK-2 cells transfected with TapSAKI-WT or TapSAKI-MUT and miR-NC mimic or miR-205 mimic. (c) The expression of miR-205 by qRT-PCR in HK-2 cells transfected with si-NC or si-TapSAKI. *P < 0.05.

To further understand the mechanism by which TapSAKI knockdown relieved LPS-induced cytotoxicity, we performed a detailed analysis for the miRNAs that potentially bind to TapSAKI. *In silico* prediction by LncBase Predicted v.2 software revealed a putative complementary sequence for miR-205 in TapSAKI (Figure 4a). To confirm this, TapSAKI luciferase reporter plasmid harboring the miR-205-binding sequence or a site-directed mutant (in the target sequence) was transfected into HK-2 cells with miR-205 mimic or miR-NC mimic. Cotransfection of wild-type reporter and miR-205 mimic in cells resulted in about a 50% reduction in luciferase activity (Figure 4b). However, no change was observed in the luciferase activity of mutant-type reporters upon miR-205 overexpression (Figure 4b), indicating the validity of these binding sites for interaction. Moreover, in comparison to the negative group, miR-205 expression was remarkably increased by TapSAKI silencing (Figure 4c), suggesting that the miR-205-binding sequence was functional. All these results strongly pointed out the role of TapSAKI as a molecular sponge of miR-205.

### 3.5 miR-205 overexpression relieved LPS-induced damage in HK-2 cells

**Figure 5:**
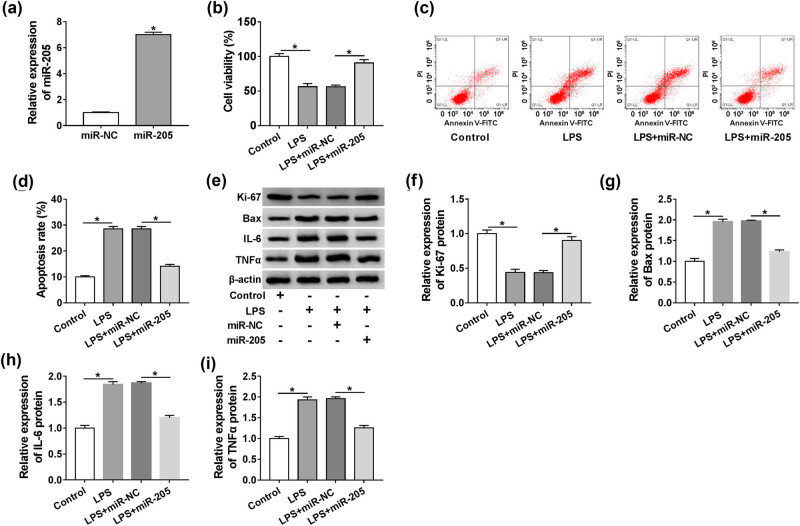
miR-205 overexpression protected HK-2 cell from LPS-induced injury. (a) qRT-PCR for miR-205 level in HK-2 cells transfected with miR-205 mimic or miR-NC mimic. CCK-8 assay for cell viability (b), flow cytometry for cell apoptosis (c and d), western blot for Ki-67, Bax, IL-6, and TNFα levels (e–i) in HK-2 cells transfected with or without miR-205 mimic or miR-NC mimic before LPS exposure. *P < 0.05.

Next, we tested the role of miR-205 in LPS-induced HK-2 cell damage. Transient introduction of miR-205 mimic, but not the miR-NC control, led to about a seven-fold augment in miR-205 expression (Figure 5a). The results of CCK-8 and flow cytometry showed that miR-205 upregulation resulted in enhanced cell viability (Figure 5b) and attenuated cell apoptosis (Figure 5c and d), which were altered by LPS stimulation. Furthermore, high miR-205 expression triggered a distinct increase in Ki-67 expression and the clear decrease in Bax, IL-6, and TNFα levels in LPS-treated HK-2 cells (Figure 5e–i). These data together hinted that miR-205 upregulation alleviated HK-2 cell injury induced by LPS.

### 3.6 miR-205 mediated the alleviated effect of TapSAKI silencing on LPS-induced HK-2 cell injury

**Figure 6:**
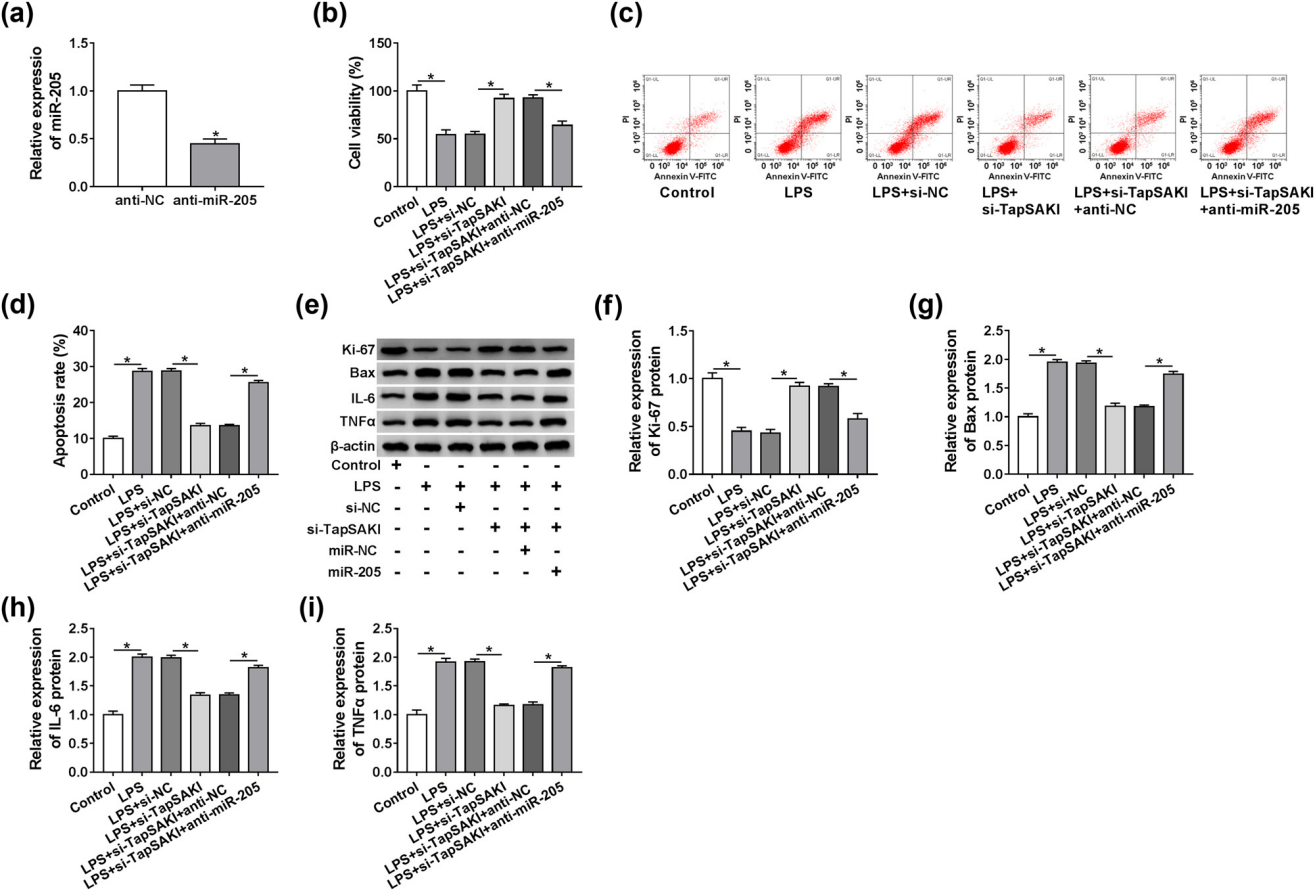
The alleviated effect of TapSAKI depletion on LPS-induced HK-2 cell damage was reversed by anti-miR-205 cotransfection. (a) miR-205 expression by qRT-PCR in HK-2 cells transfected with anti-NC or anti-miR-205. Cell viability by CCK-8 assay (b), cell apoptosis by flow cytometry (c), Ki-67, Bax, IL-6, and TNFα levels by western blot (d–h) in HK-2 cells transfected with or without si-NC, si-TapSAKI, si-TapSAKI + anti-NC, or si-TapSAKI + anti-miR-205 before LPS treatment. *P < 0.05.

To provide further mechanistic insight into the link between TapSAKI and miR-205 in LPS-induced cytotoxicity, HK-2 cells were cotransfected with si-TapSAKI and anti-miR-205 before LPS exposure. In contrast to the negative group, anti-miR-205 introduction induced about a 55% downregulation in miR-205 expression (Figure 6a), indicating the high transfection efficiency. Subsequent experiments showed that compared with the negative control, TapSAKI knockdown-mediated pro-viability (Figure 6b) and anti-apoptosis (Figure 6c) effects in LPS-induced HK-2 cells were markedly abolished by the cotransfection of anti-miR-205. Moreover, TapSAKI silencing-mediated Ki-67 augment, Bax reduction, IL-6 decrease, and TNFα repression were significantly reversed by anti-miR-205 cotransfection (Figure 6d–h). All these data strongly established a notion that TapSAKI silencing alleviated LPS-induced HK-2 cell damage by upregulating miR-205.

### 3.7 IRF3 signaling pathway was involved in the regulation of the TapSAKI/miR-205 axis on LPS-induced HK-2 cell injury

**Figure 7:**
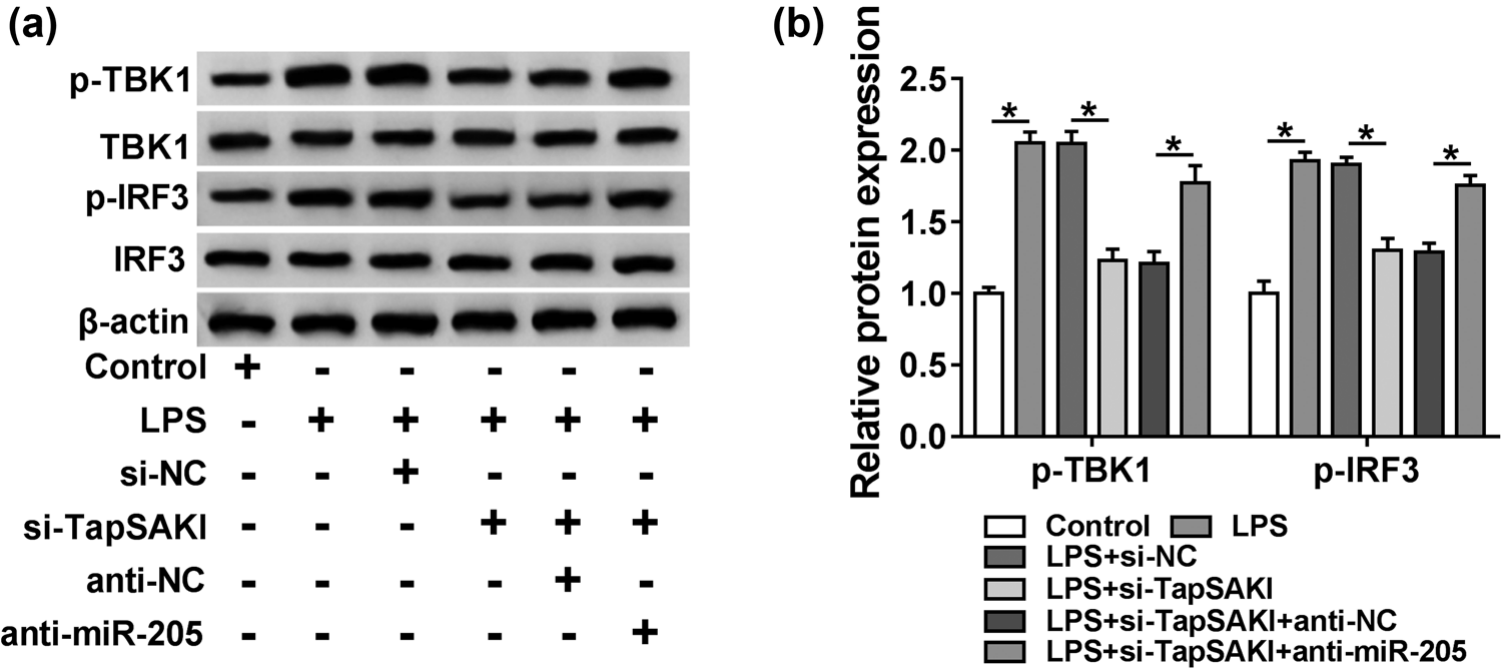
TapSAKI knockdown blocked IRF3 signaling in LPS-induced HK-2 cells via miR-205. (a and b) The expression levels of p-TBK1, TBK1, p-IRF3, and IRF3 by western blot in HK-2 cells transfected with or without si-NC, si-TapSAKI, si-TapSAKI + anti-NC, or si-TapSAKI + anti-miR-205 before LPS treatment. *P < 0.05.

IRF3 signaling, one component of the TLR4 signaling pathway, has been highlighted to contribute to sepsis pathogenesis [15,16]. TBK1 is an essential component of the IRF3 signaling pathway [17]. Hence, we investigated whether this pathway was involved in the TapSAKI/miR-205-mediated regulation on LPS-induced HK-2 cell damage by detecting the levels of p-TBK1 and p-IRF3. The data of western blot showed that when compared with the negative control, LPS induced the activation of the pathway, as evidenced by the increase of p-TBK1 and p-IRF3 expression in HK-2 cells (Figure 7a and b). Of interest, si-TapSAKI transfection resulted in reduced p-TBK1 and p-IRF3 levels in LPS-treated HK-2 cells (Figure 7a and b). However, this effect was prominently abolished by anti-miR-205 cotransfection (Figure 7a and b). Taken together, these results suggested that TapSAKI knockdown blocked IRF3 signaling in LPS-induced HK-2 cells via miR-205.

## 4 Discussion

Severe sepsis and septic shock are common and lethal syndromes [2]. The pathogenesis of sepsis is complex, and a better understanding of the pathogenesis is imperative for improving this disease treatment. LPS can induce the development of cellular inflammation and apoptosis [18]. LPS has been widely used for the establishment of the sepsis model *in vitro* and *in vivo* [19,20]. In the current work, we first established the sepsis *in vitro* model, and our data validated that LPS-induced HK-2 cell damage through weakening cell viability and promoting cell apoptosis and inflammation, in line with previous studies [21,22]. Subsequently, we further investigated the role and mechanism of TapSAKI in cell viability, apoptosis, and inflammation in LPS-induced HK-2 cells.

Dysregulation of lncRNAs is being found to have relevance to human sepsis, offering a possibility for lncRNAs as this disease diagnosis, treatment, and prognosis [4]. Among these sepsis-related lncRNAs, elevated TapSAKI level has been uncovered as a predictor of mortality in patients with acute kidney injury [14,23], which is often caused by sepsis [24]. In the current study, our data demonstrated a significant upregulation of TapSAKI in LPS-treated HK-2 cells, consistent with a recent document [8]. Moreover, we uncovered that TapSAKI silencing alleviated LPS-induced HK-2 cell injury, as evidenced by the enhancement in cell viability, as well as the suppression in cell apoptosis and inflammation.

Then, we used online software LncBase Predicted v.2 to help identify the miRNAs that potentially bind to TapSAKI. Among these predicted candidates, miR-205 was of particular interest in the current work, because it had been demonstrated to be the most downregulated miRNA in sepsis-induced acute kidney injury [12]. miR-205 has been reported to implicate in the tumorigenesis and progression of many human cancers, such as renal cell carcinoma, breast cancer, and colorectal cancer [25,26,27]. Moreover, decreased miR-205 level was associated with inflammatory breast cancer [28]. Additionally, low miR-205 expression in cutaneous T-cell lymphomas was able to distinguish between benign and malignant inflammation with the high specificity and sensitivity [29]. In the current project, we were first to identify that TapSAKI acted as a miR-205 sponge by directly binding to miR-205. Furthermore, we first uncovered that high expression of miR-205 protected HK-2 cells from LPS-induced damage. More importantly, our data first substantiated that the alleviated impact of TapSAKI silencing on LPS-induced HK-2 cell damage was mediated by miR-205.

TLR4 signaling pathway, including TLR4/NF-κB and TLR4/IRF3 pathways, contributes to the pathogenesis of sepsis [30,31], and the signaling is activated by LPS stimulation [32]. Wendy et al. reported that IRF3 signaling was activated in macrophages cultured with live bacteria and enhanced systemic inflammatory response [15]. In the present work, we first demonstrated that TapSAKI knockdown could block IRF3 signaling in LPS-induced HK-2 cells via miR-205. Similar to our findings, Shen and colleagues showed that TapSAKI enhanced inflammation damage in HK-2 cells through sponging miR-22 and regulating phosphatase and tensin homolog (PTEN)/TLR4/NF-κB pathway [8]. The current work was limited to *in vitro* studies, and more *in vivo* research studies using the sepsis model will be carried out in further work.

In conclusion, the current research suggested that TapSAKI deficiency alleviated LPS-induced injury in HK-2 cells possibly through functioning as a miR-205 sponge and regulating the IRF3 signaling pathway. Our present work provided a novel understanding of sepsis pathogenesis and a promising target for this disease treatment.

## Abbreviations

AKI: acute kidney injury
IRF3: interferon regulatory factor 3
lncRNA: long non-coding RNA
LPS: lipopolysaccharide
TapSAKI: transcript predicting survival in AKI
